# Two Single Nucleotide Deletions in the *ABCD1* Gene Causing Distinct Phenotypes of X-Linked Adrenoleukodystrophy

**DOI:** 10.3390/ijms24065957

**Published:** 2023-03-22

**Authors:** Katrin A. Dohr, Silvija Tokic, Magdalena Gastager-Ehgartner, Tatjana Stojakovic, Miroslav Dumic, Barbara Plecko, Katja K. Dumic

**Affiliations:** 1 Research Unit of Analytical Mass Spectrometry, Cell Biology and Biochemistry of Inborn Errors of Metabolism, Department of Paediatrics and Adolescent Medicine, Medical University of Graz, 8036 Graz, Austria; 2 Clinical Institute of Medical and Chemical Laboratory Diagnostics, University Hospital Graz, 8036 Graz, Austria; 3 Department of Paediatric Endocrinology and Diabetes, Clinical Hospital Centre Zagreb, 10000 Zagreb, Croatia; 4 Division of General Paediatrics, Department of Paediatrics and Adolescent Medicine, Medical University of Graz, 8036 Graz, Austria

**Keywords:** X-linked adrenoleukodystrophy, adrenomyeloneuropathy, pathogenic variant

## Abstract

X-linked adrenoleukodystrophy (X-ALD) is a rare inborn error of the peroxisomal metabolism caused by pathologic variants in the ATP-binding cassette transporter type D, member 1 (*ABCD1*) gene located on the X-chromosome. ABCD1 protein, also known as adrenoleukodystrophy protein, is responsible for transport of the very long chain fatty acids (VLCFA) from cytoplasm into the peroxisomes. Therefore, altered function or lack of the ABCD1 protein leads to accumulation of VLCFA in various tissues and blood plasma leading to either rapidly progressive leukodystrophy (cerebral ALD), progressive adrenomyeloneuropathy (AMN), or isolated primary adrenal insufficiency (Addison’s disease). We report two distinct single nucleotide deletions in the *ABCD1* gene, c.253delC [p.Arg85Glyfs*18] in exon 1, leading to both cerebral ALD and to AMN phenotype in one family, and c.1275delA [p.Phe426Leufs*15] in exon 4, leading to AMN and primary adrenal insufficiency in a second family. For the latter variant, we demonstrate reduced mRNA expression and a complete absence of the ABCD1 protein in PBMC. Distinct mRNA and protein expression in the index patient and heterozygous carriers does not associate with VLCFA concentration in plasma, which is in line with the absence of genotype–phenotype correlation in X-ALD.

## 1. Introduction

X-linked adrenoleukodystrophy (X-ALD, OMIM #300100) is the most common peroxisomal disorder caused by variants in the *ABCD1* (ATP-binding cassette, subfamily D, member 1) gene. The *ABCD1* gene, located on the Xq28, contains ten exons and encodes the ABCD1 protein, originally also termed adrenoleukodystrophy protein (ALP). ALP is a part of the ATP-binding cassette transporter superfamily and is responsible for import of very long chain fatty acids (VLCFA) into peroxisomes for β-oxidation [1]. Lack or dysfunction of the ABCD1 transporter causes accumulation of VLCFA in various tissues in the body and in blood [2,3]. The accumulation of VLCFA in the central nervous system leads to a progressive neuropathic disorder with a diverse disease phenotype in affected males [4,5]. A vast spectrum of neurologic symptoms is associated with X-ALD and no known genotypic–phenotypic correlation currently exists [6,7,8]. The most severe is the earliest onset form of childhood cerebral X-adrenoleucodystrophy (X-ALD). It is associated with progressive behavioral changes, followed by rapid motor and cognitive decline, and if untreated, death occurs within years of symptom onset [9]. Adrenomyeloneuropathy (AMN) manifests in adulthood predominantly with spinal cord involvement, progressive spasticity, weakness, neuropathies and loss of bowel and bladder control. This form is of variable severity and can also affect female carriers as well [10]. Due to additional VLCFA accumulation in the adrenal cortex, many males are affected by primary adrenal insufficiency (Addison’s disease) [11].

The overall incidence of X-ALD is estimated at 1:20,000 in the male population [12], but novel data from US newborn screening report an even higher 1:10,500 incidence rate [13].

On a molecular level, accumulated VLCFA cause disease symptoms by generating oxidative stress and inflammation, leading to macrophage activation [14] and dysfunctional peroxisomes [15], but the contribution of individual *ABCD1* variants is unknown. More than 800 *ABCD1* pathogenic and likely pathogenic variants are cataloged in the Human Gene Mutation Database [7] and in the specific ALD Variant Database [8] (available online at https://adrenoleukodystrophy.info/mutations-and-variants-in-abcd1, accessed on 15 February 2023). Further genotype–phenotype association studies are warranted for better variant interpretation, especially in the light of newborn screening, and are relevant for family counseling.

In this study, we report two unpublished pathogenic variants in the *ABCD1* gene: (i) Variant c.253delC [p.Arg85Glyfs*18] in exon 1, found in a 10-year-old boy with progressive cerebral X-ALD phenotype and his 17-year old brother with an early onset AMN phenotype; and (ii) Variant c.1275delA [p.Phe426Leufs*15] in exon 4, diagnosed in a 26-year-old male leading to AMN and in his 6-year-old nephew with primary adrenal insufficiency. The variant (c.1275delA [p.Phe426Leufs*15]) is characterized on mRNA and protein level in the index patient and in other family members.

## 2. Results

### 2.1. Clinical Characteristics of the Index Patient in Family 1 with Cerebral X-ALD and His Affected Older Brother with AMN

**Figure 1:**
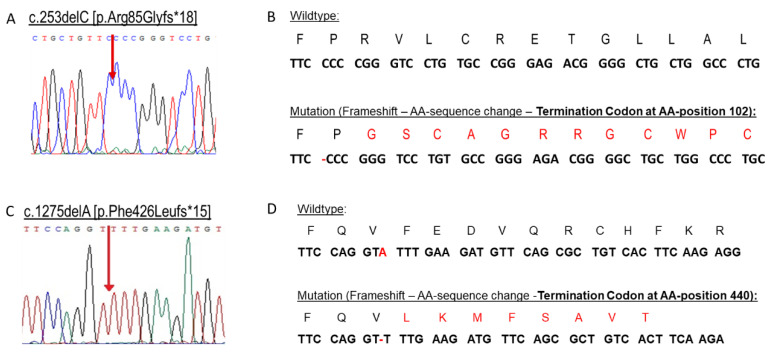
Novel single nucleotide deletions in the *ABCD1* gene. An electropherogram after Sanger sequencing showing a sequence change in the index patient from family 1 (**A**) and from family 2 (**C**) and the resulting amino acid sequence change (**B**) and (**D**), respectively.

The patient was the second son of healthy, unrelated parents with a pedigree that was empty for neurologic disorders. His mother was entirely healthy at age 36 years. He presented with impaired hearing at age 8 years. Over the following months, he developed rapidly progressive ataxia, loss of vision and speech and became wheelchair bound. Cranial MRI showed bilateral increased signal intensity of occipital lobes, with involvement of the corpus callosum, accompanied by symmetric gadolinium enhancement, and was initially suspected for encephalitis, followed by cortisone treatment without beneficial effect. When referred for a second opinion at age 10, a definite diagnosis was still pending. Skin pigmentation was normal. He had poor social interaction with no reaction to sounds and no tracking of objects. Pupils were enlarged with no reaction to light. There was marked generalized spasticity with lack of voluntary movements. A control cMRI showed markedly progressed leukodystrophy, involving frontal lobes and subcortical areas. A suspected diagnosis of X-ALD was confirmed with elevated C26, C24, and an elevated ratio of C24/22 and C26/22 (Table 1), and by genetic testing (Figure 1). Basal ACTH was increased to 3378 pg/mL (n.v. 50 pg/mL) while cortisol was unaffected (521 nmol/L, n.v. 201–681 nmol/L), indicating latent adrenal insufficiency. He was started on oral hydrocortisone, 20 mg/m^2^/day, and was otherwise treated under palliative care.

The older brother of the index case in family 1 showed first symptoms at age 16 years with gait disturbance and infrequent stumbling that slowly progressed over the following years. When seen at age 18 years, he had bronze colored skin, intact cranial nerve function, mild ataxia worsening upon eye closure, hyperreflexia of his lower limbs, and a positive Babinski sign. Vibration sense was slightly reduced and there was bilateral distal hypoesthesia of the lower limbs. His SARA Score was 2. Bladder and bowel continence was normal. Cognition was intact. Cranial MRI and MRS were normal, including normal NAA concentrations in the center semiovale. Nerve conduction velocity of peroneal nerves was reduced to 26.9 m/sec (n > 45). Auditory evoked potentials showed bilateral prolonged latency. Visual evoked potentials were normal. VLCFA in plasma were elevated (Table 2). Basic ACTH was increased to 3752 pg/mL (n.v. 50 pg/mL) while cortisol was 219 nmol/L (n.v. 201–681 nmol/L). As with his brother, he was started on oral hydrocortisone, 20 mg/m^2^/day, and was unfortunately lost to follow-up.

### 2.2. Genetic Confirmation of X-ALD in Family 1

Genetic analysis revealed a single nucleotide deletion c.253delC [p.Arg85Glyfs*18] in exon 1 leading to a frameshift mutation and an early stop codon (Figure 1A,B). According to the in silico prediction tools, this variant leads to nonsense-mediated mRNA decay and the complete absence of the ABCD1 protein. It is predicted as pathogenic with high probability (Table 2). The occurrence of the mutation in 119 wild types was 0 (Figure S1).

### 2.3. Clinical Characteristics of the Index Patient in Family 2 with AMN and His Young Nephew with Addison’s Disease

**Figure 2:**
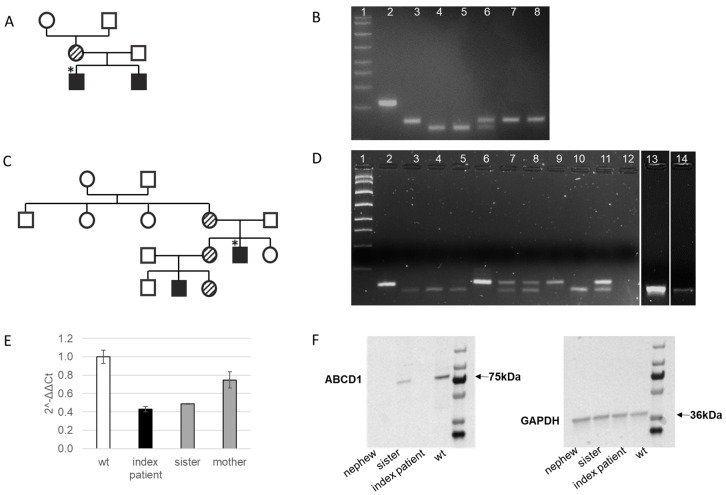
Pedigree analysis of the c.253delC variant (**A**) performed by a restriction digest (**B**) and for c.1275delA novel variant (**C**,**D**). *ABCD1* mRNA expression in the index patient and heterozygous sister and mother in comparison to wild type WT. Expression of GAPDH was used for normalization (**E**). Immunoblot of the index patient and heterozygous sister in comparison to control; GAPDH was used as a loading control (**F**). Lines on the gel in (**B**): (1) standard, (2) PCR product, (3) WT, (4) Index patient, (5) affected brother, (6) mother (heterozygous), (7) grandmother (WT), grandfather (WT). Lines on the gel in (**D**): (1) standard, (2) PCR product, (3–5) WT, (6) Index patient, (7) sister (heterozygous), (8) mother (heterozygous), (9) nephew (affected), (10) nephew (WT), (11) niece (heterozygous), (12) no template control, (13) father (WT), (14) sister (WT). * Indicates the index patient in the pedigree.

The patient is 26-year-old with AMN. He had experienced the occasional morning nausea and anorexia and had noticed progressive pigmentation of the skin from around the age of 17 years. At the age of 20 years, he developed an adrenal crisis and subsequently, he was diagnosed with Addison’s disease (cortisol 27 nmol/L, n.v. 150–680 nmol/L; ACTH > 440.4 pmol/L, n.v. 2–14 nmol/L, PRA 53 µg/L/h, n.v. 0.2–2.8 µg/L/h). Further evaluation also revealed primary hypothyroidism due to autoimmune thyroid disease and hypergonadotropic hypogonadism, which led to erroneous diagnosis of autoimmune polyglandular syndrome type 2. Treatment with hydrocortisone, fludrocortisone, levothyroxine and testosterone was introduced. In the following years, he started developing progressive neurological symptoms including spastic paraparesis and erectile dysfunction despite normal testosterone concentrations on substitution. Additionally, he had scanty scalp hair and gynecomastia. Brain MRI was normal while spinal cord MRI revealed diffuse atrophy of thoracic spinal cord and hyperintensive lesions of posterior columns of C2 and C3 segments on T2 weighted images. Diagnosis of AMN was established at the age of 26 years after measuring elevated plasma concentration of VLCFA C26, C24 and elevated ratio of C24/22 and C26/22 (Table 1). AMN was further confirmed by genetic testing. Analysis of family members (Figure 2A) revealed three heterozygous asymptomatic female carriers of the mutation (mother, sister and her daughter) and a 6-year-old affected nephew with latent adrenal insufficiency (cortisol 132 nmol/L, n.v. 150–680 nmol/L; ACTH 78 pmol/L, n.v. 2–14 nmol/L, PRA 5.4 µg/L/h, n.v. 0.2–2.8 µg/L/h) and completely normal clinical and neurological status. He was immediately put on replacement treatment with hydrocortisone and fludrocortisone to avoid adrenal crisis.

### 2.4. Genetic Confirmation of AMN in the Index Patient in Familiy 2 and Family Members Evaluation

After increased C24 and C26, VLCFA, as well as the C24/C22 and C26/C22 ratios being measured in the index patient´s plasma (Table 1), we proceeded to genetic analysis by Sanger Sequencing. In genomic DNA of the index patient, a likely pathogenic variant c.1275delA [p.Phe426Leufs*15] was detected in exon 4 of the *ABCD1* gene (Figure 1B,D). This hitherto unknown variant, as in family 1, also leads to a frameshift in the open reading frame of the *ABCD1* gene, resulting in an erroneous amino acid sequence and subsequently, an early termination through an early stop codon, which may lead to nonsense-mediated mRNA decay (Figure 1C,D). According to the in silico prediction tools this variant is predicted as pathogenic with high probability (Table 2). The occurrence of c.1275delA variant in 119 wild types was 0 (Figure S2).

We further analyzed plasma VLCFA concentration in family members. Increased C24 and C26 VLCFA, as well as the C24/C22 ratio, were found also in plasma of the mother, sister and two of her children (boy and a girl). The C26/C22 ratio was increased only in the index patient and in the affected nephew (Table 1). Restriction analysis revealed concordant results with plasma VLCFA concentrations for hemizygously affected nephew and heterozygous female carriers, namely mother of the index patient, older sister and her daughter (Figure 2D).

### 2.5. Molecular Characterization of the Novel Frameshift Variant c.1275delA [p.Phe426Leufs*15]

To confirm if the novel single nucleotide deletion indeed leads to nonsense-mediated mRNA decay, we analyzed the *ABCD1* mRNA expression in isolated PBMC from the index patient and heterozygous female carriers, sister, and mother. In comparison to wild type, mRNA expression was reduced by 57% in the index patient and by 51% and 25% in his sister and mother, respectively (Figure 2E). We further analyzed ABCD1 protein expression in the index patient and his affected nephew, as well in his sister. Immunoblot analysis revealed no expression of the ABCD1 protein either in the index patient or in the affected nephew. The sister of the index patient, a heterozygous female carrier, showed 35% expression in comparison to wild type (Figure 2F). These results support the proposed prediction of a nonsense-mediated mRNA decay.

## 3. Discussion

Phenotypes in male X-ALD patients include cerebral X-ALD (childhood, adolescent and adult), adrenomyeloneuropathy (AMN) with or without cerebral involvement, and Addison´s disease only. Female carriers may develop symptoms involving myelopathy and peripheral neuropathy. Initial phenotypic presentation of X-ALD is variable and evolution over time is likely [4]. We have identified two novel pathogenic *ABCD1* variants, small deletions in exon 1 and exon 4, respectively. Both were predicted to lead to a nonsense-mediated mRNA decay and absence of a functional protein but lead to distinct phenotypes in index patients and family members.

Pathogenic variant c.253delC led to the most severe X-ALD phenotype in the index patient in family 1, namely, childhood cerebral X-ALD, which included loss of vision and speech and the patient was wheelchair bound within 2 years of symptom onset. In contrast, his older brother developed a rare, juvenile form of AMN. Pedigree analysis revealed their completely asymptomatic mother as a carrier with both grandparents being wild types. This could be in line with a de novo mutation in the paternal sperms of the maternal grandfather.

The index patient of family 2 (c.1275delA) was initially diagnosed as having autoimmune polyglandular syndrome type 2. Progressive neurological symptoms developed over time and led to reevaluation of his initial diagnosis, VLCFA measurement and final diagnosis of X-AMN. Biochemical and molecular evaluation of family members revealed one new patient, a 6-year-old nephew with latent adrenal insufficiency. It also revealed three completely asymptomatic female carriers—mother, sister and niece.

We here report two novel single nucleotide deletions, c.253delC [p.Arg85Glyfs*18] and c.1275delA [p.Phe426Leufs*15], in the *ABCD1* gene, both leading to a frameshift variant and a premature termination codon at 102 and 440 amino acid sequence, respectively. The occurrence of a premature termination codon triggers a well-known cellular surveillance function and mRNA degradation to prevent synthesis of a truncated protein [16,17]. Moreover, for the c.1275delA [p.Phe426Leufs*15], we have shown low *ABCD1* mRNA, and a complete absence of the ABCD1 protein in the index patient and his affected nephew. Interestingly, the sister of the index patient, a heterozygous carrier, as revealed by restriction analysis and increased VLCFA profiles, displayed similar *ABCD1* mRNA levels to the index patient, but showed ABCD1 protein synthesis. Even this low amount of protein was sufficient to carry out normal function, as all heterozygous carriers in the family, namely, the mother, sister and niece of the index patient, were completely asymptomatic.

**Figure 3:**
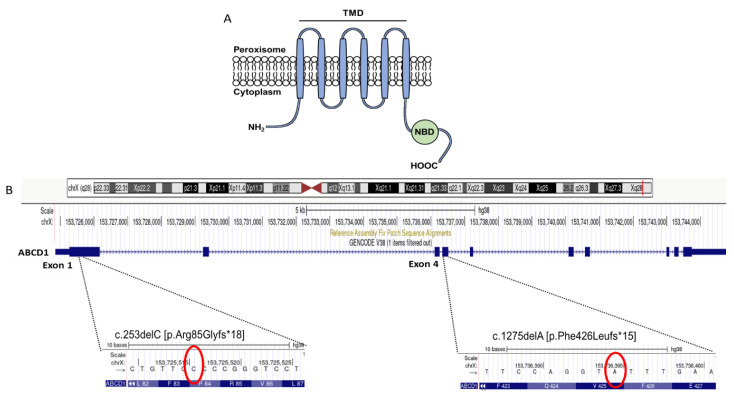
Product of the *ABCD1* gene encodes a transmembrane protein composed of a transmembrane domain (TMD) and a nucleotide binding domain (NBD) responsible for import of VLFCA from cytosol into peroxisomes (**A**). Chromosomal location of the novel frameshift mutations c.253delC in exon 1 and c.1275delA in exon 4 in the *ABCD1* gene on the X-choromosome (**B**).

**Figure 4:**
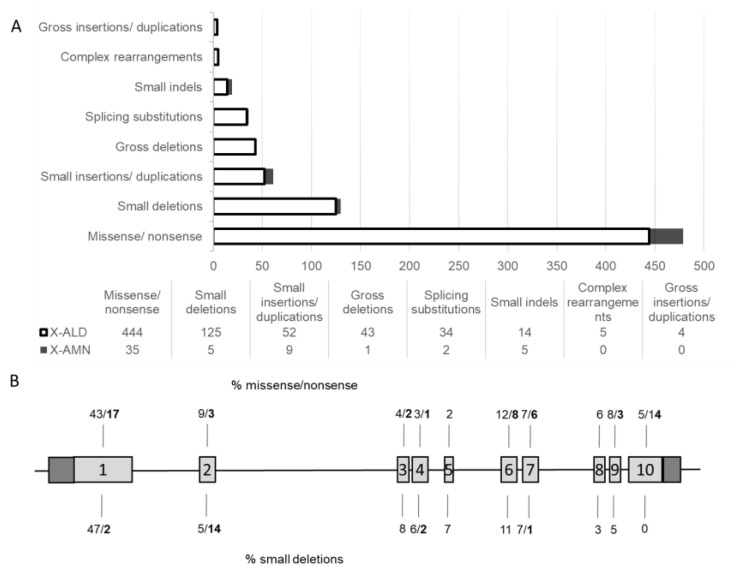
Schematic representation of all *ABCD1* variants currently listed in the Human Gene Mutation Database [7] based on type of the reported gene variant (**A**), on gene location (**B**), and on disease phenotype (**A**,**B**). In B, the numbers in bold represent percentage of the rare AMN phenotype in the two most common variant types, missense/nonsense and small deletions.

ABCD1 protein is encoded by ten exons, out of which exons 1 and 2 encode the transmembrane domain (TMD) responsible for substrate specificity while exons 5-10 encode the nucleotide binding domain (NBD) that binds ATP (Figure 3A). Our two variants are located in exon 1 and exon 4, respectively (Figure 3B), and without activation of the nonsense-mediated mRNA decay, their translation would lead to a truncated protein lacking most of the TMD and a whole NBD in c.253delC, and a complete absence of the NBD in c.1275delA. Within the *ABCD1* gene most common types of sequence changes are missense/nonsense variants and small deletions (Figure 4A) accounting for 43% and 47% of changes in exon 1, respectively (Figure 4B). To date, only 6% of the small deletions have been pinpointed to exon 4, out of which only 2% associate with the milder AMN phenotype [7].

Despite improvements in medical care, diagnostics and bioinformatic tools, a correlation between patient’s molecular genotype and phenotype, as well as disease progression, is still pending. One single variant may lead to all three main phenotypes in male patients [18,19,20]. Although both pathogenic variants reported here were predicted to cause a complete absence of the ABCD1 protein, the variant c.253delC in exon 1 led to the most severe cerebral X-ALD, while c.1275delA in exon 4 caused moderate AMN phenotype. The culprit seems to be multifactorial and besides toxicity of the accumulated VLCFA, modifier genes and environmental/epigenetic factors might contribute [21,22,23].

The VLCFA profile measurement is a very accurate tool for diagnosis of X-ALD and should be performed in every male with Addison´s disease even in the absence of neurological symptoms. Diagnosis can further be confirmed by genetic testing. Family studies are essential. Through them, female carriers can be diagnosed. The epigenetic phenomenon of X-inactivation may contribute to variable clinical presentation in females with X-linked conditions, such as X-ALD [24]. Some female carriers may develop similar symptoms to ones found in males affected with adrenomyeloneuropathy; therefore, they should be clinically evaluated and followed [25,26].

Evaluation of family members can also reveal male patients in the presymptomatic phase of the disease. A lack of all clinical components of the disease at the initial presentation can lead to late diagnosis of X-ALD. Treatment is available in some cases, such as allogeneic HCT in the early stages of cerebral ALD and substitutional therapy for adrenocortical insufficiency. Recognition and early diagnosis are critical, improve outcome and can avoid premature deaths of affected males. Preconceptional and prenatal testing should be offered to families at risk.

## 4. Methods

### 4.1. Plasma VLFCA Measurements

Very long chain fatty acids (VLCFA) were measured via gas chromatography/mass spectrometry as described previously [27].

### 4.2. DNA Isolation

Genomic DNA was isolated and purified from 500 µL EDTA-whole blood samples using the QiAmp^®^DNA Blood Mini Kit (Qiagen). The samples were prepared according to the manufacturer’s protocol using DNase free water for elution [28].

### 4.3. Sequencing and Restriction Analysis

M13-tailed primer pairs were designed to amplify and sequence the complete coding region of the *ABCD1* gene of the index patient, including 100 bp of the intron–exon boundaries (Table S1). Additionally, a primer pair (*ABCD1* exon 8-9) was designed to selectively amplify exon 8 and exon 9, since it was difficult to cut out the interference of so-called pseudogenes which have a 92–96% similarity to this region [29]. The PCR was performed using the AmpliTaq Gold™ DNA Polymerase with Buffer II, MgCl_2_ (Thermo Fisher, Vilnius, Lithuania) and DMSO (Sigma-Aldrich, Steinheim, Germany) and for exons 8–10 GoTaq^®^ Polymerase (Promega, Madison, WI, USA). Amplicons were separated on a 2% E-Gel™ EX Gel (Invitrogen^TM^) and DNA bands were visualized with iBright™ CL750 Imaging System (Thermo Fisher Scientific).

The sequencing was performed via Sanger Sequencing at the Institute for Human Genetics, Medical University of Graz, using BigDye^®^ Cycle Sequencing with the BigDye^®^ Terminator v1.1 Cycle Sequencing Kit (Applied Biosystems^TM^).

For the pathogenic variant c.253delC in the index patient and analysis in all other family members, restriction digest was performed using a mismatch primer (5′-CTCCTGCGGCTGCTGTTCCgC-3′) to introduce a recognition sequence for the endonuclease MvnI (5′-CGCG-3′, Roche) in the mutant allele. A PCR using the mismatch and the ABCD1 E1R-primer was performed using GoTaq^®^ Polymerase (Promega) to amplify the region of interest and implement the mismatch. If the allele contains the wildtype sequence and the mismatch is incorporated, MvnI cuts only at one recognition site, leading to two amplicons of 149 bp and 70 bp. However, if the mutation is present and the mismatch is incorporated, the 149 bp amplicon includes an additional recognition sequence of MvnI. Therefore, the endonuclease cuts the amplicon at two recognition sides, resulting in three shortened amplicons of 129 bp, 70 bp and 20 bp. In case of the c.1275delA, the restriction digest was performed using a mismatch primer (5′-CAGCGCTGAACATCTTCAAg-3′) to introduce a recognition sequence for the endonuclease RsaI (5′-GTAC-3′, New England Biolabs) in the wildtype. A PCR using the *ABCD1* Exon 4F primer and the mismatch primer was performed to amplify the region of interest and implement the mismatch. If the mutation is present, the amplicon does not include the recognition sequence for RSaI, even if the mismatch is incorporated and the amplicon remains at its initial length of 134 bp. However, if the allele contains the wildtype sequence and the mismatch, RsaI cuts the amplicon at its recognition side, leading to two shortened amplicons of 113 bp and 21 bp. To visualize the size of these amplicons, a gel electrophoresis was performed using a 4% agarose gel and a 3.5% agarose gel, respectively. This method was also used to determine whether the pathogenic variants were present in 119 people not suffering from X-ALD to determine the frequency of the alleles.

### 4.4. RT-qPCR

mRNA from whole blood was isolated from specific *PAXgene Blood RNA Tubes* containing RNase inhibitors and the *PAXgene Blood RNA Kit V2* (PreAnalytiX) was used according to the manufacturer’s protocol. The High-Capacity RNA-to-cDNA^TM^ Kit (Applied Biosystems^TM^) was used to transcribe mRNA to cDNA. RT-qPCR was performed using Fast SYBR^TM^ Green Master Mix (Applied Biosystems^TM^, Vilnius, Lithuania) and 10ng of cDNA in triplicates for each primer pair (10 µM, Table S2). The expression fold–change was determined using the ΔΔCT-method [19].

### 4.5. Western Blot

Protein was isolated from leukocytes, which were collected from EDTA-whole blood. The isolation was carried out using RIPA buffer (Sigma-Aldrich) and short sonification intervals to increase the yield. Protein concentration was determined using Pierce™ BCA Protein Assay Kit (Thermo Scientific, Vilnius, Lithuania). Per sample, 15 µg of protein were separated via SDS-Page using NuPAGE™ 4–12%, Bis-Tris, 1.5 mm, Mini Protein Gel (Invitrogen^TM^). The protein was then transferred onto a nitrocellulose membrane with 0.45 µm pores (Invitrogen^TM^). To check the transfer, the membrane was stained with Ponceau S. As a loading control, GAPDH was used (# sc-32233 Santa Cruz; 1:1000). Primary ABCD1 antibody was used from Invitrogen (# MA5-26606, 1:1000). Anti-mouse HRP-conjugated secondary antibodies (Cell Signaling Technologies, 1:10,000) were used and the detection was performed using Amersham™ ECL Prime Western Blotting Detection Kit.

## 5. Conclusions

Despite extensive research, the underlying pathology of X-ALD remains poorly understood. The lack of reliable disease models has been a significant obstacle in X-ALD studies. As a result, the mechanisms that contribute to disease phenotype and symptom progression are still unclear.

## Figures and Tables

**Table 1:** VLCFA measurements in the index patients and family members carrying either a variant c.253delC [p.Arg85Glyfs*18] in exon 1 or c.1275delA [p.Phe426Leufs*15] in exon 4. In bold are values that exceed the reference values.

	Phenotype	C22	C24	C26	C24/C22	C26/C22
Reference Range:		11.2–48.7	7.10–28.9	0.18–0.48	0.68–0.98	0.007–0.03
Family 1. (c.253delC)						
Index Case (hemizygous)	X-ALD	26.1	44.70	1.97	1.70	0.070
Brother (hemizygous)	AMN	14.4	26.40	1.06	1.80	0.070
Family 2. (c.1275delA)						
Index Case (hemizygous)	AMN	25.70	49.30	1.68	1.92	0.065
Mother (heterozygous)	asymptomatic	35.4	39.00	0.74	1.10	0.021
Father (wild type)		20.6	15.9	0.14	0.77	0.007
Sister (wild type)		27	25.5	0.19	0.94	0.007
Sister (heterozygous)	asymptomatic	24.6	29.4	0.56	1.2	0.023
Nephew (hemizygous)	Addison´s	20.8	36.6	1.10	1.76	0.053
Nephew (wild type)		19.8	21.2	0.16	1.07	0.008
Niece (heterozygous)	asymptomatic	26.2	36.9	0.65	1.41	0.025

**Table 2:** Novel single nucleotide deletions in the *ABCD1* gene identified in this study and the results of the in silico prediction tools.

Physical Location	Variant	Type	Mutation Taster	SIFT	Protein Length
chr23:152990974delC	c.253delC	Frameshift	damaging (LOF: 0.98; tree vote: 190|10)	damaging (score: 0.858)	NMD
chr23:153001849delA	c.1275delA	Frameshift	damaging (LOF: 0.98; tree vote: 186|14)	damaging (score: 0.858)	NMD

Reference genome: GCRh37.13, *ABCD1* transcript NM_000033.4. LOF = loss of function. NMD = nonsense-mediated mRNA decay.

## Data Availability

All data are provided in the manuscript and available upon contacting the corresponding author.

## Supplementary Materials

The following supporting information can be downloaded at: https://www.mdpi.com/article/10.3390/ijms24065957/s1.
