# Supplementary material for: Two Single Nucleotide Deletions in the ABCD1 Gene Causing Distinct Phenotypes of X-Linked Adrenoleukodystrophy
Source: Int J Mol Sci. 2023 Mar 22;24(6):5957. doi: 10.3390/ijms24065957 (PMC10051867; doi:10.3390/ijms24065957)
Supplement: Supplementary file 1 [file ijms-24-05957-s001.zip › ijms-2168579-supplementary.pdf]

Supplementary Material to:

Two Single Nucleotide Deletions in the *ABCD1* Gene Causing Distinct Phenotypes of X-Linked Adrenoleukodystrophy

Supplementary Table S1. M13-tag primer for Sanger Sequencing of ABCD1 gene.

Name	Sequence
ABCD1_1F_M13	**CACGACGTTGTAAAACGAC**CATCGCAAGGTTTCCAGTT
ABCD1_1R_M13	**GGATAACAATTTCACACAGG**TGGCACTGTTGACGAAGGTA
ABCD1_1AF_M13	**CACGACGTTGTAAAACGAC**GCACCTTCCTGTCGGTGTAT
ABCD1_1AR_M13	**GGATAACAATTTCACACAGGC**ACACGCGAGTGCATGTAG
ABCD1_1BF_M13	**CACGACGTTGTAAAACGAC**CGTGGCTGTGACTTCCTACA
ABCD1_1BR_M13	**GGATAACAATTTCACACAGG**CAGCTCTAAGGCCAGGACAA
ABCD1_2F_M13	**CACGACGTTGTAAAACGAC**CCACCCAATCGTAACCTCTG
ABCD1_2R_M13	**GGATAACAATTTCACACAGG**CTGTGCCTGGAGAAGTGACA
ABCD1_3F_M13	**CACGACGTTGTAAAACGAC**GATGTGCTCTGGGTTGGTTT
ABCD1_3R_M13	**GGATAACAATTTCACACAGG**ATAGGGAGGGGACAGCCTTA
ABCD1_4F_M13	**CACGACGTTGTAAAACGAC**CATCCTTGCCATGCTTCTCT
ABCD1_4R_M13	**GGATAACAATTTCACACAGG**GCAGCTACTGTCTGGGAAGG
ABCD1_5F_M13	**CACGACGTTGTAAAACGAC**GACACTGGGGGAAGAGTTCA
ABCD1_5R_M13	**GGATAACAATTTCACACAGG**TTAGGAGTGTGGGGAGTTGG
ABCD1_6F_M13	**CACGACGTTGTAAAACGAC**CTCTCAAGGCTGGTCAGGAG
ABCD1_6R_M13	**GGATAACAATTTCACACAGG**CAGATCCAAAACAGGGCAGT
ABCD1_7F_M13	**CACGACGTTGTAAAACGAC**CTGCCCTGTTTTGGATCTGT
ABCD1_7R_M13	**GGATAACAATTTCACACAGG**CCATCTGTGTGGTGTTGGTC
ABCD1_8F_M13	**CACGACGTTGTAAAACGAC**GGCTTGGACTCCACCGTATC
ABCD1_8R_M13	**GGATAACAATTTCACACAGG**GCTCAGGCTCCACTGAGCC
ABCD1_9F_M13	**CACGACGTTGTAAAACGAC**TGGGTGCTGGTGGAACTGA
ABCD1_9R_M13	**GGATAACAATTTCACACAGG**CTGCTGATGACAGCCGCCT
ABCD1_10F_M13	**CACGACGTTGTAAAACGAC**AGGCGGCTGTCATCAGCA
ABCD1_10R_M13	**GGATAACAATTTCACACAGG**CGGGAAGGGTTTTCTAGGAGG

The M13-tag sequence is depicted in bold.

Supplementary Table S2. Primer pairs used for RT-qPCR.

Name	Sequence
ABCD1 exon 1_forward	CCTACTACCGGGTCAGCAAC
ABCD1 exon 1_reverse	GAGTAGAGGTGGGCCACAGA
ABCD1 exon 4_forward	GTGCACGAGATGTTCCAGGT
ABCD1 exon 4_reverse	CGGCCTATGGTCCCAGAC
GAPDH_forward	AAGGTCATCCCTGAGCTGAA
GAPDH_reverse	TTTGGCAGGTTTTTCTAGACG

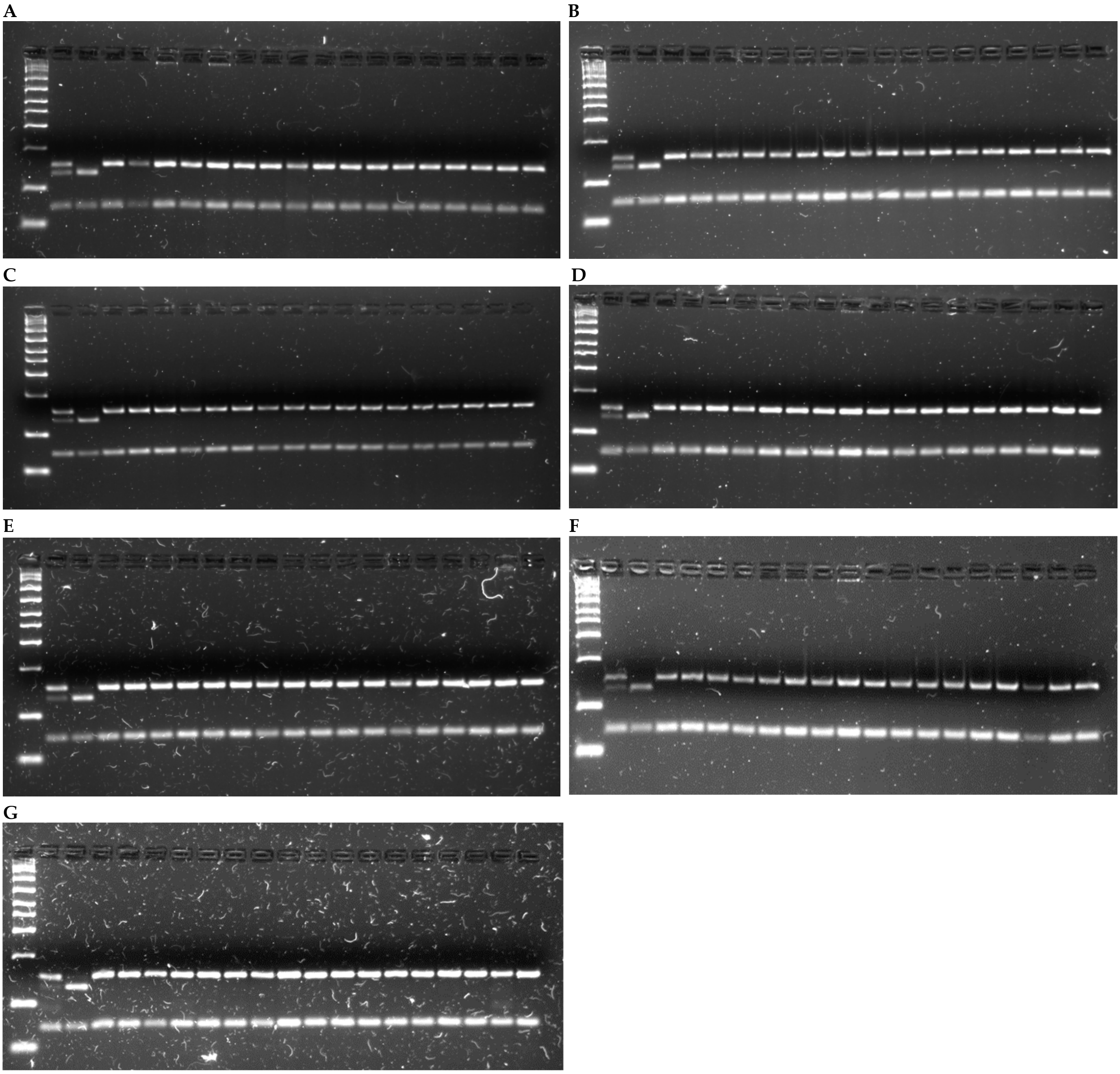

Figure S1: Frequency determination of the variant c.253delC in 119 people not suffering from X-ALD. (**A**) (1) standard, (2) mother (heterozygous), (3) index patient (hemizygous), (4-20) wildtype female. (**B**) (1) standard, (2) mother (heterozygous), (3) index patient (hemizygous), (4-20) wildtype female. (**C**) (1) standard, (2) mother (heterozygous), (3) index patient (hemizygous), (4-20) wildtype female. (**D**) (1) standard, (2) mother (heterozygous), (3) index patient (hemizygous), (4-12) wildtype female, (13-20) wildtype male. (**E**) (1) standard, (2) mother (heterozygous), (3) index patient (hemizygous), (4-20) wildtype male. (**F**) (1) standard, (2) mother (heterozygous), (3) index patient (hemizygous), (4-20) wildtype male. (**G**) (1) standard, (2) mother (heterozygous), (3) index patient (hemizygous), (4-20) wildtype male.

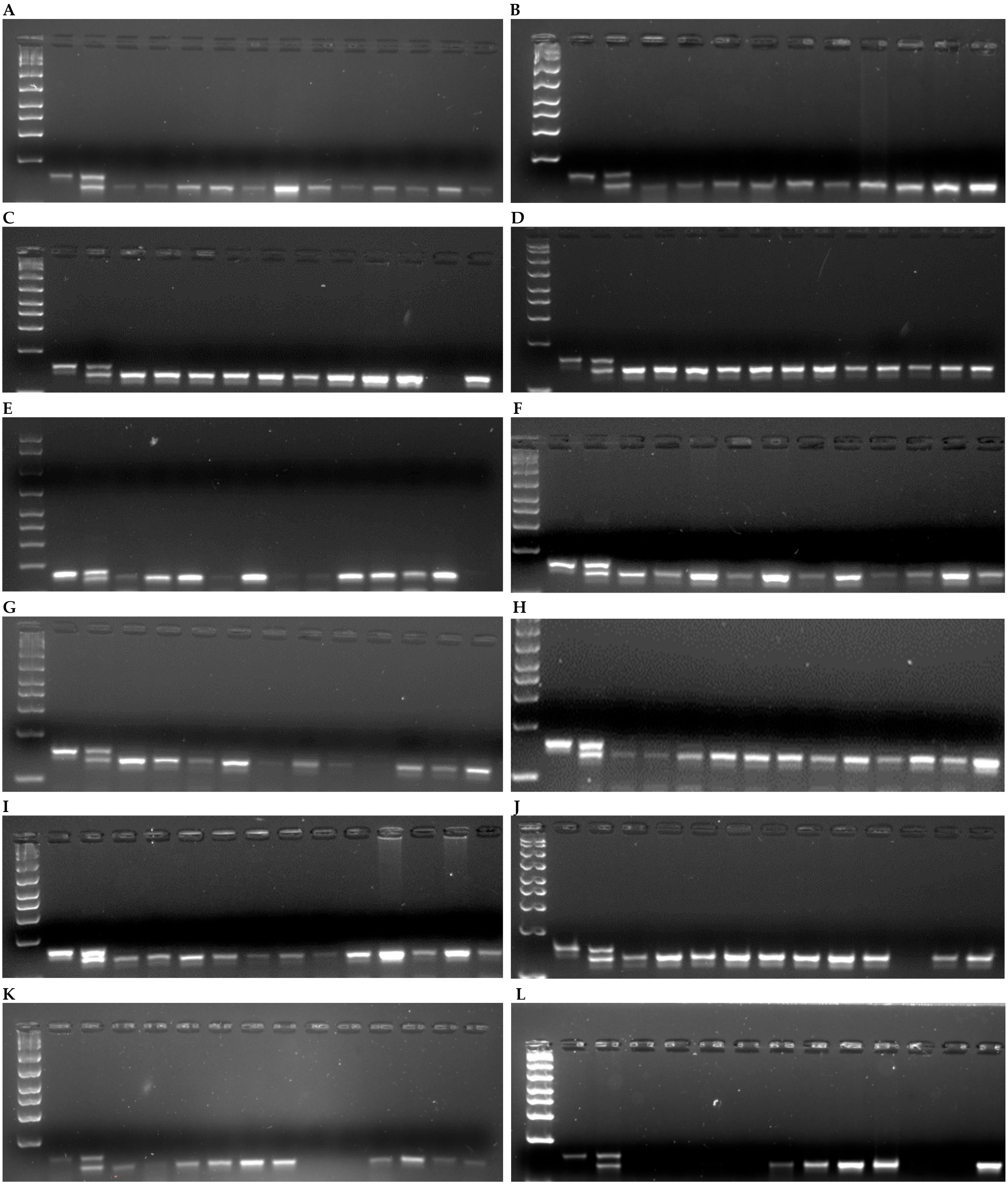

Figure S2: Frequency determination of the variant c.1275delA in 119 people not suffering from X-ALD. (**A**) (1) standard, (2) index patient (hemizygous), (3) sister (heterozygous), (4-15) wildtype male. (**B**) (1) standard, (2) index patient (hemizygous), (3) sister (heterozygous), (4-13) wildtype male. (**C**) (1) standard, (2) index patient (hemizygous), (3) sister (heterozygous), (4-12, 14) wildtype female. (**D**) (1) standard, (2) index patient (hemizygous), (3) sister (heterozygous), (4-15) wildtype male. (**E**) (1) standard, (2) index patient (hemizygous), (3) sister (heterozygous), (4-6,8,11-14) wildtype female. (**F**) (1) standard, (2) index patient (hemizygous), (3) sister (heterozygous), (4-14) wildtype male. (**G**) (1) standard, (2) index patient (hemizygous), (3) sister (heterozygous), (4-7, 12-14) wildtype female, (9) wildtype male. (**H**) (1) standard, (2) index patient (hemizygous), (3) sister (heterozygous), (4-12) wildtype male, (13-15) wildtype female. (**I**) (1) standard, (2) index patient (hemizygous), (3) sister (heterozygous), (4, 6-11) wildtype female, (5, 12-15) wildtype male. (**J**) (1) standard, (2) index patient (hemizygous), (3) sister (heterozygous), (4-11, 13-14) wildtype female. (**K**) (1) standard, (2) index patient (hemizygous), (3) sister (heterozygous), (4, 6-9,12-15) wildtype male. (**L**) (1) standard, (2) index patient (hemizygous), (3) sister (heterozygous), (8-11, 14) wildtype female.
